# Naltrexone as an Antipruritic Agent for Surgical Scar Pruritus

**DOI:** 10.7759/cureus.105611

**Published:** 2026-03-21

**Authors:** Manuela Florez, Alexander Baron, Anjali Nirmalani-Gandhy, Glenn Catalano

**Affiliations:** 1 Mental Health and Behavioral Sciences, James A. Haley Veterans' Hospital, Tampa, USA; 2 Psychiatry and Behavioral Neurosciences, University of South Florida Morsani College of Medicine, Tampa, USA

**Keywords:** blepharoplasty, naltrexone, neuropsychiatric conditions, opioid antagonist, pruritus, surgical scar pruritus

## Abstract

Naltrexone is a competitive opioid receptor antagonist that is FDA-approved for the treatment of alcohol and opioid use disorders. It is also used as an off-label treatment for a plethora of conditions spanning multiple specialties, including multiple sclerosis, chronic pain, Crohn’s disease, and compulsive disorders. Naltrexone has also been used off-label for the treatment of pruritus in patients with burn injuries, atopic dermatitis, cholestatic dermatitis, chronic urticaria, and immunotherapy-induced pruritus. Naltrexone is thought to alleviate pruritus through its antagonist effect on the µ-opioid receptor at pain-transmitting and pruritoceptive dorsal horn neurons. This case report explores the treatment of persistent surgical scar pruritus following endoscopic brow lift and bilateral upper eyelid blepharoplasties under general anesthesia. After trialing first-line treatments of oral antihistamines and topical antipruritics, naltrexone was initiated. The treatment dose was titrated between 25 and 100 mg daily, and the pruritus is currently well-controlled with a dose of 25 mg twice a day. While our case report supports the limited available data for the use of naltrexone as treatment for pruritus in certain conditions, more research is warranted to further establish efficacy, safety, and specific diagnosis indications. Naltrexone is not currently FDA-approved to treat pruritus.

## Introduction

Naltrexone is primarily used for the treatment of alcohol and opioid use dependence. It is an opioid receptor antagonist, which reduces cravings and the pleasurable effects associated with drug or alcohol use. While the exact process by which naltrexone reduces cravings is not fully understood, it is thought to do so in part by blocking μ-opioid receptors (MOR) on gamma-aminobutyric acid (GABA) interneurons in the ventral tegmental area (VTA) of the brain. Normally, GABA interneurons inhibit dopaminergic neurons, limiting dopamine release. When opioids bind to receptors on GABA interneurons, they reduce this inhibitory effect, allowing increased dopamine release. By blocking MOR on GABA interneurons, naltrexone prevents this opioid-induced disinhibition, thereby modulating dopamine signaling in the mesolimbic system [1].

Although naltrexone is not typically used for the treatment of pruritus, there is research that suggests its potential benefits in certain cases. Several studies and case reports have suggested that naltrexone can reduce itching in certain conditions associated with endogenous opioids, such as cholestatic pruritus, uremic pruritus, eczema senilis, cutaneous lymphoma, immunotherapy-induced pruritus, post-burn pruritus, and chronic psychogenic pruritus [2-7]. Naltrexone's opioid-blocking property is thought to be potentially helpful in these specific situations, as opioids are known to induce or intensify pruritus [4,8]. The mechanism through which opioids cause pruritus has been explained via the effect of opioids on pain-transmitting and pruritoceptive dorsal horn neurons, which run parallel to each other in the dorsal horn of the spinal cord [4,8]. Opioids act on opioid-sensitive interneurons between these neurons to induce both analgesia and itch. Conversely, naltrexone acts on these interneurons to diminish the sensation of pruritus [4,8].

At present, there is a limited amount of literature on naltrexone for pruritus, and to our knowledge, there are no reports on the use of naltrexone for the treatment of post-surgical scar pruritus. The goal of this case report is to describe the successful use of naltrexone in a post-browpexy patient with surgical scar pruritus.

## Case presentation

**Figure 1:**
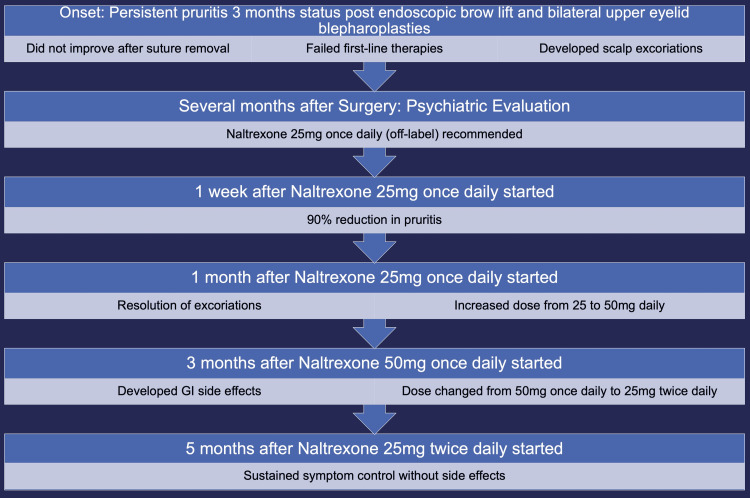
Clinical timeline of post-surgical pruritus and response to naltrexone This figure outlines the clinical course of persistent scalp pruritus following brow lift and blepharoplasty, refractory to first-line therapies. Initiation of naltrexone resulted in rapid improvement with a dose-dependent response, recurrence upon discontinuation, and sustained symptom control after dose adjustment to 25 mg twice daily. Image was created using Microsoft PowerPoint (Microsoft® Corp., Redmond, WA).

The patient is a 65-year-old male who has been followed with an outpatient psychiatrist for management of dysthymia, generalized anxiety disorder, a full-body tic that worsens with anxiety, claustrophobia, and chronic pain syndrome. The patient underwent an endoscopic brow lift and bilateral upper eyelid blepharoplasties for visual field impairment secondary to brow ptosis and upper eyelid dermatochalasis. He received general endotracheal anesthesia during the procedure. The procedure was completed with no complications. Following the surgery, he had mild pain and a vague sensation of itchiness above his right eye. The pain quickly resolved; however, the itching sensation progressed to affect his entire scalp over the course of two weeks. A few months after the procedure, the patient underwent a non-operative procedure to remove the suture over his right brow under local anesthesia with 0.25% bupivacaine, and the procedure was completed without complications. The suture removal did not reduce or intensify the itching that he had developed. He tried using loratadine 10 mg daily and prescription triamcinolone acetonide 0.1% cream twice a day to help control the itching of his head without any improvement. Because of the constant itching, he began to develop excoriations on his head. Several months later, he met with his outpatient psychiatrist and discussed his concern over the persistent itching. Given the timing of his symptoms after the procedure and an identifiable organic cause, he was diagnosed with post-surgical scar pruritus rather than psychogenic pruritus, a diagnosis of exclusion made when no organic cause for pruritus was present [7]. The patient was prescribed naltrexone 25 mg as an off-label medication that had shown efficacy for non-surgical pruritic disorders in the literature review. Shortly after that appointment, the patient had difficulty with compliance due to an unrelated illness. Once he recovered, he resumed taking naltrexone 25 mg daily, and he very quickly found relief from the pruritus. One week after starting the naltrexone therapy, he reported a subjective 90% reduction in itching, and by one month, the lesions on his head from where he had been itching had resolved. He developed some mild constipation from using naltrexone but denied any other associated adverse effects. At that visit, he discussed using a higher dose to fully resolve the itching, and naltrexone was increased to 50 mg daily. One month after being on the naltrexone 50 mg, the itching resolved completely. Notably, when he ran out of medication for two days, the itching came back but resolved again once he restarted the medication. Three months after starting the 50 mg dose, the patient developed increased GI side effects and stopped the medication. The itching returned, and he had restarted at the 25 mg dose when he was seen for follow-up. At the next appointment, he continued to endorse itching at the 25 mg dose, so his dose was increased to 25 mg twice a day for better GI tolerability. After five months, the patient remains on a daily dose of 25 mg twice a day of naltrexone without significant side effects, and notes continued control of itchiness on the medication (Figure 1).

## Discussion

This case demonstrates a novel use of naltrexone for the treatment of surgical scar pruritus, an expansion upon its current off-label use as an anti-pruritic in other clinical conditions. The treatment was selected after the patient did not respond to more traditional antipruritic agents such as antihistamines. Naltrexone was specifically selected based on the literature evidence of naltrexone’s effectiveness for the treatment of pruritus. Naltrexone has evidence from randomized control trials, albeit with limited sample size (N with a range of 16-52), in addition to observational studies and case reports that support its clinical efficacy as an antipruritic agent used predominantly in dermatology for cholestatic pruritus, atopic dermatitis, and chronic urticaria [4]. 

Naltrexone is commonly used in psychiatry and is currently FDA-approved for treating alcohol use disorder and opioid use disorder. It acts as a competitive antagonist at opioid receptors. For both alcohol use disorder and opioid use disorder, its action as an opioid antagonist reduces levels of euphoric intoxication and dependence [9]. In addition to this effect, naltrexone is thought to decrease the hypothalamic-pituitary-adrenal axis’s response, decreasing the negative effect of alcohol withdrawal [10]. Naltrexone also has some off-label uses for other neuropsychiatric conditions, including trichotillomania, fibromyalgia, complex regional pain syndrome, Hailey-Hailey disease, and multiple sclerosis [2,11]. At higher doses (50-100 mg), naltrexone blocks opioid receptors on GABA neurons in the VTA, which inhibits reinforcing dopaminergic pathways [11]. This is thought to be the mechanism behind treating compulsive behaviors such as gambling, self-harm behaviors, alcoholism, eating disorders, kleptomania, and trichotillomania [11]. At lower doses (1-5 mg), naltrexone acts as an immunomodulator on toll-like receptors, proinflammatory cytokines, T-cell proliferation, and expression of chemokine receptors and adhesion molecules through transient blockade of opioid receptors [12,13]. This is thought to be the mechanism behind treating fibromyalgia, chronic pain, Hailey-Hailey disease, and multiple sclerosis [12,13].

While the efficacy of naltrexone specifically for post-surgical scar pruritus has not been researched, there are several studies and case reports that show the efficacy of naltrexone for the treatment of pruritus in a variety of medical conditions. An observational study found that naltrexone can be used as an alternative treatment for severe pruritus in elderly patients where pruritus was not controlled by anti-histamines alone [4]. The study found that cholestatic pruritus, uremic pruritus, eczema senilis, cutaneous lymphoma, prurigo nodularis, xerotic eczema, and pruritus of unknown origin all responded to naltrexone at a range of doses (25-150 mg) but most commonly studied at the 50 mg dose, benefiting our patient. [4]. There have been at least two case reports that have demonstrated naltrexone’s success at a 50 mg dose in managing immunotherapy-induced pruritus resistant to steroids [5]. Naltrexone has also been shown to have a positive impact on pruritus in burn patients and chronic psychogenic pruritus [6,7].

The proposed mechanism for naltrexone’s antipruritic properties is based on its antagonism at the MOR [4,8]. Endogenous and exogenous opioids induce or intensify pruritus; therefore, the reversal of this effect by an opioid antagonist can diminish pruritus. Pain-transmitting and pruritoceptive dorsal horn neurons are parallel to each other in the dorsal horn of the spinal cord and are connected by interneurons that are opioid sensitive [4,8]. Opioids act on these pain-transmitting neurons and interneurons to produce analgesia. At the same time, their suppression of the opioid-sensitive interneurons connected to the pruritoceptive neurons stimulates the itching sensation [4,8]. Inversely, naltrexone’s pharmacologic blockade of the MOR on the interneurons diminishes the sensation of itch by suppressing transmission of the pruritoceptive neurons [4,8].

This article is limited in scope as a single case report; however, it seeks to contribute an additional piece of evidence toward the efficacy of naltrexone as an anti-pruritic. Alternative etiologies, including psychogenic and neuropathic pruritus, were considered given the patient’s psychiatric comorbidities and symptom chronicity. However, the temporal association with surgery, initial localization to the operative site, and subsequent progression pattern support a post-surgical etiology. Another limitation of this case is the reliance solely on subjective feedback, which limits the objective quantification of the treatment response. Further study of naltrexone would allow for a better understanding of the potential efficacy of and its role in the management of post-surgical scar pruritus.

## Conclusions

In conclusion, to our knowledge, this represents the first reported case of naltrexone used in the treatment of post-surgical scar pruritus, extending its previously described off-label use in pruritic conditions such as cholestatic pruritus, atopic dermatitis, and chronic urticaria. Naltrexone was chosen based on emerging evidence demonstrating efficacy in treating pruritus after the patient did not respond to traditional antipruritic agents such as antihistamines. The patient reported an improvement in pruritus that was associated with a total daily dose of naltrexone 50 mg. Naltrexone, traditionally used in the treatment of alcohol and opioid use disorders, is thought to have antipruritic effects due to its antagonistic action at the MOR on interneurons connecting pain-transmitting and pruritoceptive dorsal horn neurons. While the literature on the off-label use of naltrexone as a second-line agent for the treatment of pruritus in a variety of medical conditions has been increasing, it's important to note that more research is needed to establish the efficacy, safety, and appropriate dosing of naltrexone for pruritus. Naltrexone is not currently FDA-approved to treat pruritus. 
